# The Mechanical and Electronic Properties of Carbon-Rich Silicon Carbide

**DOI:** 10.3390/ma9050333

**Published:** 2016-04-30

**Authors:** Qingyang Fan, Changchun Chai, Qun Wei, Yintang Yang

**Affiliations:** 1 Key Laboratory of Ministry of Education for Wide Band-Gap Semiconductor Materials and Devices, School of Microelectronics, Xidian University, Xi’an 710071, China; ccchai@mail.xidian.edu.cn (C.C.); ytyang@xidian.edu.cn (Y.Y.); 2 School of Physics and Optoelectronic Engineering, Xidian University, Xi’an 710071, China; qunwei@xidian.edu.cn

**Keywords:** silicon carbide, mechanical properties, electronic properties, 62.20.de, 62.20.dq, 71.20.-b

## Abstract

A systematic investigation of structural, mechanical, anisotropic, and electronic properties of SiC_2_ and SiC_4_ at ambient pressure using the density functional theory with generalized gradient approximation is reported in this work. Mechanical properties, *i.e.*, the elastic constants and elastic modulus, have been successfully obtained. The anisotropy calculations show that SiC_2_ and SiC_4_ are both anisotropic materials. The features in the electronic band structures of SiC_2_ and SiC_4_ are analyzed in detail. The biggest difference between SiC_2_ and SiC_4_ lies in the universal elastic anisotropy index and band gap. SiC_2_ has a small universal elastic anisotropy index value of 0.07, while SiC_2_ has a much larger universal elastic anisotropy index value of 0.21, indicating its considerable anisotropy compared with SiC_2_. Electronic structures of SiC_2_ and SiC_4_ are calculated by using hybrid functional HSE06. The calculated results show that SiC_2_ is an indirect band gap semiconductor, while SiC_4_ is a quasi-direct band gap semiconductor.

## 1. Introduction

Silicon carbide has been investigated since 1907, when Captain H. J. Round first found that silicon carbide can be used as a material for making light-emitting diodes and detectors in early radios [1,2]. SiC is a candidate of choice for high-speed, high-temperature, high-power, and high-frequency device applications because of its wonderful physical properties and electronic properties, such as wide bandgaps, high saturated electron drift velocities, high thermal conductivities, and high-breakdown electric fields. Furthermore, SiC is hard, chemically stable, and resistant to radiation damage. In addition to these extraordinary mechanical properties, SiC is also highly resistant to irradiation, which makes this material a first-choice candidate for various nuclear applications, such as a structural material in future fusion reactors [3,4]. SiC has potential applications in weighty bad circumstances due to its high chemical stability with a good resistance to corrosion. Like silicon, as a semiconductor, SiC can also be doped due to its electronic properties. Moreover, SiC is used in high-power and high-temperature devices. The combination of all these mechanical, electrical, and thermal properties makes SiC a highly sought-after material for biosensor applications [5].

Five independent elastic constants of 4H- and 6H-SiC single crystals have been determined via Brillouin scattering [6]. Elastic constants and sound velocities, calculated using first-principles calculations as a function of pressure, were presented for 2H-SiC by Sarasamaker *et al.* [7]. The stability and mobility of non-dissociated screw dislocations in 2H-, 4H- and 3C-SiC have been investigated using first-principles calculations. For SiC, it has in fact been shown that plasticity properties at low temperatures are mainly due to these extended defects, regarding which, very little is known. Previous optical work [8,9,10,11] on SiC has focused on the 3C and 6H polytypes because only small attention could be paid to other polytypes; however, 50-mm-thin 4H- and 6H-SiC wafers have become commercially available in recent years. The structural stability and electronic properties of the Si*_m_*C*_n_* graphyne-like monolayers with 18-, 18-, 24-graphyne type structures have been systematically studied using a transferable and reliable semi-empirical Hamiltonian by Yan *et al.* [12]; they found that the flat SiC and SiC_9_ graphyne-like monolayers have semiconductor properties with an energy gap of 0.96 eV and 0.69 eV, respectively. The slightly buckled Si_2_C_8_ graphyne-like monolayer, on the other hand, behaves like a tiny gap material.

The carbon-rich, silicon-rich, and germanium-rich binary compounds have also been investigated by using density functional theory methodology [13,14,15]. Two new phases of Si_8_C_4_ and Si_4_C_8_ with *P*4_2_/*nm* symmetry were proposed by Zhang *et al.* [15]; both Si_8_C_4_ and Si_4_C_8_ were proven to be dynamically and mechanically stable. The band structures of Si_8_C_4_ and Si_4_C_8_ indicate that they are both indirect semiconductors. Moreover, the density functional theory has also been successfully applied to predict the physical and chemical properties of some other binary compound materials, such as Ca-Mg [16], Si-Ge [17,18], and *X*Bi_3_ (where *X* = B, Al, Ga, and In) [19].

Using first-principles calculations, two new SiC_2_ and SiC_4_ phases of carbon-rich silicon carbide are proposed in this paper. We propose SiC_2_ (space group: *P*4_2_*nm*) and *t*-SiC_4_ (space group: *P*2_1_/*m*), whose structures are based on *t*-SiCN [20] and *P*2_1_/*m*-carbon [21], with Si substituting for C. In the present work, we will investigate the structural, chemical bonding, elastic, mechanical anisotropy, and electronic properties of SiC_2_ and SiC_4_.

## 2. Materials and Methods

The calculations were performed using density functional theory (DFT) [22,23], within Vanderbilt ultra-soft pseudo-potentials [24], generalized gradient approximation (GGA), in the form of Perdew–Burke–Ernzerhof (PBE) [25], PBEsol [26], and local density approximation (LDA), in the form of Ceperley and Alder data as parameterized by Perdew and Zunger (CA-PZ) [27], as implemented in the Cambridge Serial Total Energy Package (CASTEP) [28] code. C-2*s*^2^2*p*^2^ and Si-3*s*^2^3*p*^2^ were treated as valence electrons. The cut-off energy was selected as 400 eV, and the *k*-point sampling of the Brillouin zone was constructed using the Monkhorst–Pack scheme [29], with 10 × 10 × 6 and 5 × 12 × 8 grids in primitive cells of SiC_2_ and SiC_4_, respectively. The electronic properties of SiC_2_ and SiC_4_ were calculated by using the Heyd–Scuseria–Ernzerhof (HSE06) hybrid functional [30]. The equilibrium crystal structures were achieved by utilizing geometry optimization in the Broyden–Fletcher–Goldfarb–Shanno (BFGS) [31] minimization scheme. The self-consistent convergence of the total energy was 5 × 10^−6^ eV/atom; the maximum force on the atom was 0.01 eV/Å; the maximum ionic displacement was within 5 × 10^−4^ Å; and the maximum stress was within 0.02 GPa. The phonon spectra of SiC_2_ and SiC_4_ required using the linear response approach, called the density functional perturbation theory (DFPT), which is one of the most popular methods for the *ab initio* calculation of lattice dynamics [32].

## 3. Results and Discussion

**Figure 1:**
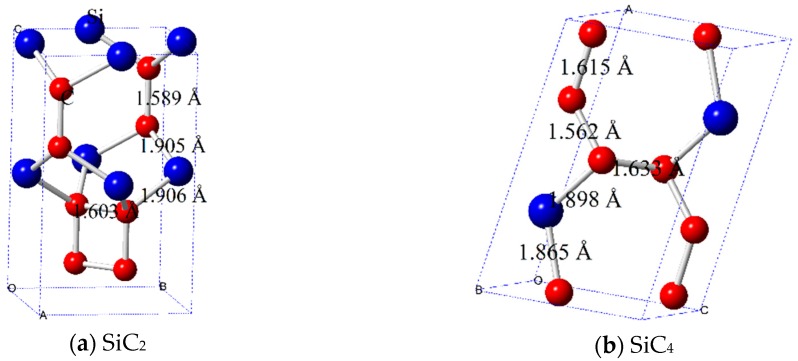
Unit cell crystal structures of SiC_2_ (**a**) and SiC_4_ (**b**).

The crystal structures of SiC_2_ and SiC_4_ are shown in Figure 1. There are 12 and 10 atoms in a conventional cell of SiC_2_ and SiC_4_, respectively. There are twelve atoms in the conventional cell of SiC_2_, with atomic positions (Fractional coordinates) of C (0.3650, 0.3650, 0.3577) and (0.3650, 0.3650, 0.1342) and Si (0, 0.5, −0.0039); there are ten atoms in the conventional cell of SiC_4_, with atomic positions (Fractional coordinates) of C (0.4862, 0.25, 0.6069), (0.7057, 0.75, 0.1069), (0.0263, 0.75, 0.4019), and (0.9484, 0.75, 0.0992) and Si (0.3015, 0.75, 0.1714). SiC_2_ has a tetragonal crystal structure, with the space group of *P*4_2_*nm* (No. 102), while SiC_4_ has a monoclinic crystal structure, with the space group of *P*2_1_/*m* (No. 11). The calculated equilibrium lattice parameters of SiC_2_ and SiC_4_ are listed in Table 1. At zero pressure, the lattice constants calculated from GGA of SiC_2_ are *a* = 4.1968 Å and *c* = 7.1067 Å, while the lattice parameters of SiC_4_ are *a* = 6.7550 Å, *b* = 2.7629 Å, *c* = 4.3794 Å, and *β* = 75.782°. The densities of SiC_2_ and SiC_4_ are 2.765 g/cm^3^ and 3.191 g/cm^3^, respectively.

**Figure 2:**
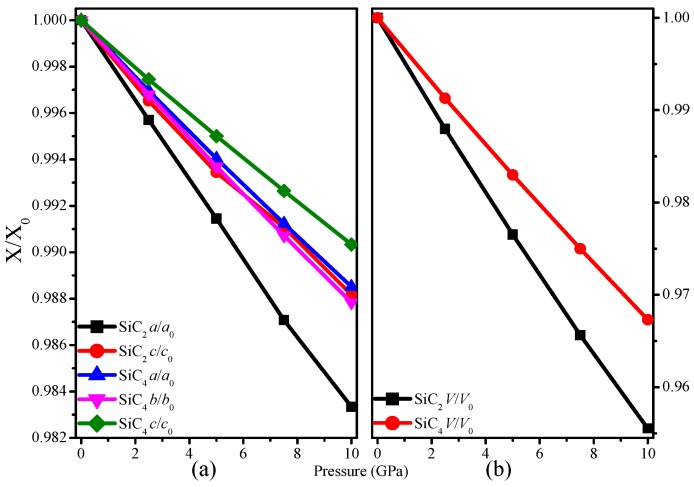
The compression lattice constants *a*/*a*_0_, *b*/*b*_0_, *c*/*c*_0_ as functions of pressure SiC_2_ (**a**) and SiC_4_ (**b**).

In Figure 2, we illustrate the pressure dependence of the equilibrium lattice parameters for SiC_2_ and SiC_4_ under pressure from 0 to 10 GPa. For SiC_2_, it can be easily observed that the compressibility along the *a*-axis (*b*-axis) is easier than along the *c*-axis. For SiC_4_, the incompressibility of the *c*-axis is slightly greater than that of the *a*-axis and *b*-axis. Figure 2b shows that the incompressibility of SiC_4_ is slightly greater than that of SiC_2_. SiC_2_ has four different bond lengths, namely, C–C bonds are 1.589 Å and 1.603 Å, while C–Si bonds are 1.905 Å and 1.906 Å. SiC_4_ has five different bond lengths, namely, C–C bonds are 1.562 Å, 1.615 Å and 1.633 Å, while C–Si bonds are 1.865 Å and 1.898 Å. The average C–C and C–Si bonds are 1.592 Å and 1.906 Å, 1.608 Å and 1.882 Å for SiC_2_ and SiC_4_, respectively. The C–C and C–Si bonds for diamond and SiC are 1.535 Å and 1.892 Å for comparison, respectively.

**Figure 3:**
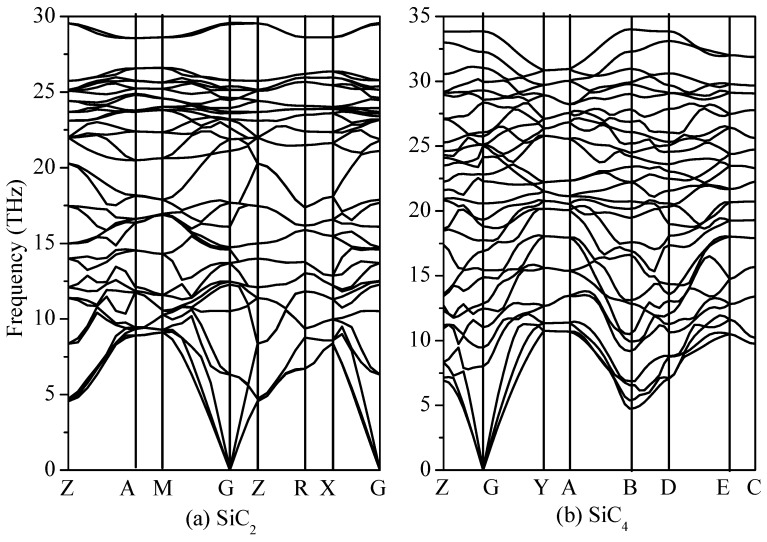
Phonon spectra for SiC_2_ (**a**) and SiC_4_ (**b**).

The elastic constant is used to describe the mechanical resistance of crystalline materials to externally applied stresses. The calculated elastic constants of SiC_2_ and SiC_4_ are shown in Table 2. From Table 2, it is evident that both SiC_2_ and SiC_4_ are mechanically stable because the elastic constants can simultaneously satisfy all of Born’s criteria for the mechanical stability of tetragonal and monoclinic symmetry [33,34]. To ensure the stability of SiC_2_ and SiC_4_, the phonon spectra are calculated at ambient pressure (0 K and 0 GPa). Figure 3 shows the phonon dispersions of SiC_2_ and SiC_4_. There is no imaginary frequency, which means that SiC_2_ and SiC_4_ are stable at ambient pressure. The elastic constants and phonon calculation have confirmed that the predicted SiC_2_ and SiC_4_ are mechanically and dynamically stable, respectively.

Using the Voigt–Reuss–Hill method [41,42,43], the bulk modulus (*B*) and shear modulus (*G*) are estimated [44,45]. Young’s modulus (*E*) and Poisson’s ratio (*ν*) are significant elastic parameters of materials; they are calculated using the formula *E* = 9*BG*/(3*B* + *G*) and *v* = (3*B* − 2*G*)/[2(3*B* + *G*)], respectively. The calculated elastic modulus and Poisson’s ratio of SiC_2_ and SiC_4_ are also shown in Table 1. For 3C-SiC, the elastic constants and elastic moduli are much closer to the experimental values; thus, we use the results within LDA to compare the big or small values of the elastic modulus. The bulk modulus, shear modulus, and Young’s modulus of SiC_4_ are greater than those of SiC_2_. The bulk modulus, shear modulus, and Young’s modulus of SiC_2_ are close to those of 3C-SiC. The Young’s modulus of SiC_4_ is much greater than that of 3C-SiC and SiC_2_. According to Pugh [46], a larger *B*/*G* value (*B*/*G* > 1.75) for a solid represents ductility, while a smaller *B*/*G* value (*B*/*G* < 1.75) usually means brittleness. The *B*/*G* values of SiC_2_ and SiC_4_ are 1.25 and 1.10, respectively. In other words, SiC_4_ is more brittle than SiC_2_. Poisson’s ratio is a factor for the degree of directionality of chemical bonds [47], being *v* = 0.1 for covalent materials and typically *v* = 0.25 for ionic materials [48]. In SiC_2_ and SiC_4_, the Poisson’s ratios are 0.18 and 0.15, respectively, suggesting the complex bond essence in SiC_2_ and SiC_4_.

Moreover, the hardness of SiC_2_ and SiC_4_ is calculated using Lyakhov and Oganov’s model [34]. The hardness of SiC_2_ and SiC_4_ is 33.6 and 44.0 GPa, respectively. These results match well with our previous prediction. Thus, SiC_2_ is a hard material, and SiC_4_ is a superhard material, with potential technological and industrial applications. The value of hardness of SiC, calculated using this model, is 29.3 GPa. The hardness of SiC_2_ and SiC_4_ is slightly greater than that of SiC because there is no C–C bond in SiC. The calculated and experimental hardness of diamond is 91.2 GPa [49] and 90.0 GPa [50], respectively, for comparison.

**Figure 4:**
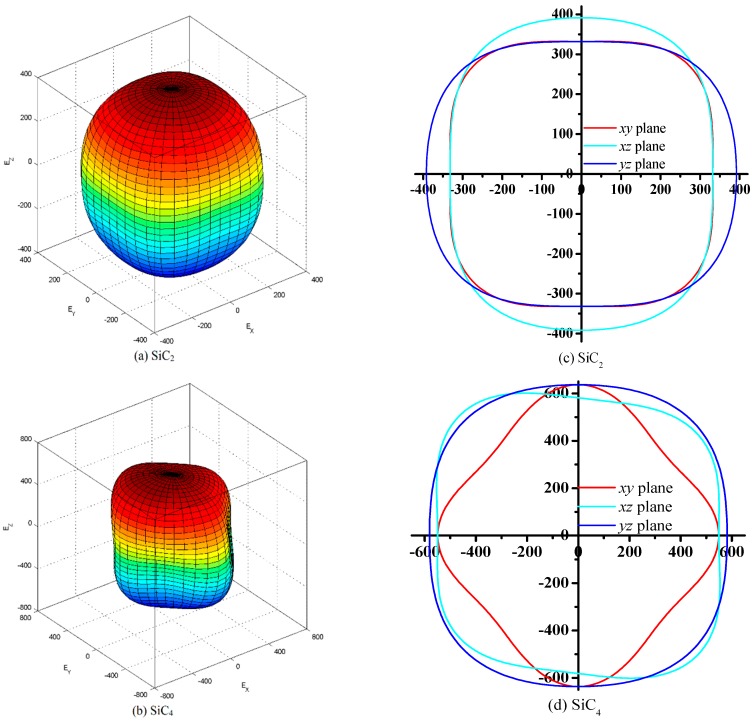
The directional dependence of Young’s modulus for SiC_2_ (**a**) and SiC_4_ (**c**); 2D representation of Young’s modulus in the *xy* plane, *xz* plane, and *yz* plane for SiC_2_ (**b**) and SiC_4_ (**d**).

Anisotropy is the property of being directionally dependent, as opposed to isotropy, which implies identical properties in all directions. It can be defined as a difference, when measured along different axes, in a material’s physical or mechanical properties. Young’s modulus for all possible directions and the 2D representation of Young’s modulus in the *xy*, *xz*, and *yz* planes for SiC_2_ and SiC_4_ are shown in Figure 4a–d, respectively. For an isotropic system, the 3D directional dependence would show a spherical shape, while the deviation degree from the spherical shape reflects the content of anisotropy [51]. The Young’s modulus of SiC_2_ varies between 332 and 411 GPa; for SiC_4_, Young’s modulus varies between 476 and 688 GPa. The ratios of *E*_max_ and *E*_min_ are 1.24 and 1.45 for SiC_2_ and SiC_4_, respectively. SiC4 exhibits a larger anisotropy in its Young’s modulus than that of SiC_2_. Another way of measuring the elastic anisotropy is given by the universal anisotropic index (*A*^U^), which is defined as *A*^U^ = 5*G*_V_/*G*_R_ + *B*_V_/*B*_R_ − 6, where *B* and *G* denote the bulk modulus and shear modulus, respectively, and the subscripts V and R represent the Voigt and Reuss approximations, respectively. Moreover, there must be *A*^U^ greater than or equal to zero; for isotropic materials, *A*^U^ must be equal to zero. The *A*^U^ of SiC_2_ is 0.07, which shows that SiC_2_ exhibits a smaller anisotropy; for SiC_4_, the larger *A*^U^ (0.21) shows a larger anisotropy.

**Figure 5:**
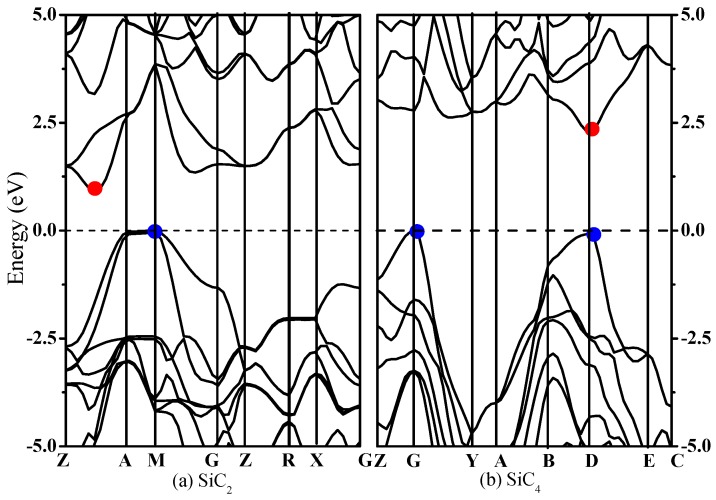
Electronic band structures of SiC_2_ (**a**) and SiC_4_ (**b**). The red and blue points indicate the conduction band minimum and valence band maximum, respectively.

**Figure 6:**
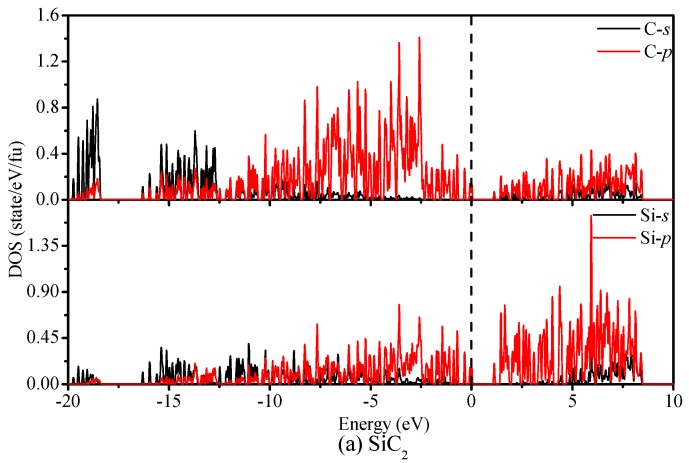

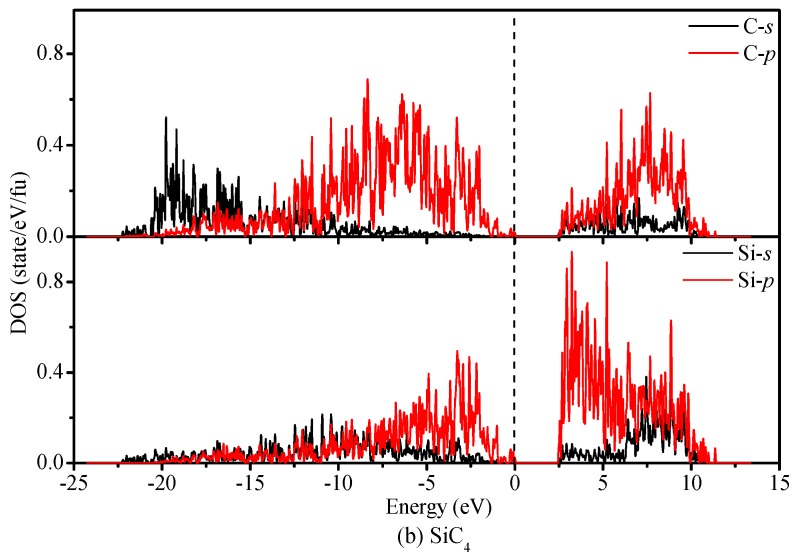
The partial density of states of SiC_2_ (**a**) and SiC_4_ (**b**).

It is well known that the electronic structure determines the fundamental physical and chemical properties of materials. The failure of LDA and GGA to accurately predict the band gaps of semiconducting materials is caused by a functional derivative discontinuity of the exchange–correlation potential, which can be avoided by using the hybrid functional. Thus, we calculate the band structure and density of states (DOS) of SiC_2_ and SiC_4_ by using the HSE06 functional, which are illustrated in Figure 5. From Figure 5, we can easily find that SiC_2_ and SiC_4_ are semiconductors with a band gap of 0.91 eV and 2.28 eV, respectively. For SiC_2_, the conduction band minimum (CBM) is at (0.2353 0.2353 0.5000) (Fractional coordinates) along the Z–A direction, while the valence band maximum (VBM) is located at (0.5000 0.5000 0.0714) along the A–M direction (see Figure 5a). For SiC_4_, CBM is at the D point, while the VBM is located at the G point (see Figure 5b). The direct gap at D is 2.34 eV, which is slightly larger than the indirect gap of 2.28 eV. Thus, SiC_4_ has a quasi-direct band gap. Figure 6a shows the partial density of state (PDOS) of SiC_2_; the PDOS is divided into three parts: the first is the energy range from −18 eV to −10 eV, where the contribution from Si-*p* is very small compared with that of other orbitals, and the main contributions to the upper band are from the C-*s* orbital. The middle band is in the range from –10 eV to 0 eV; the main contributions in this part are from the C-*p* orbital and Si-*p* orbital. The last band has energies above the Fermi level. In the upper band, the contribution from the Si-*p* orbital is great compared with that of other orbitals for the first place, while for the second, the contribution from the C-*p* orbital is great. From Figure 6b, we find that the PDOS of SiC_4_ is similar to that of SiC_2_. For the energy range from −25 eV to −15 eV, the contribution from C-*s* is very great compared with that from the other orbitals. For the energy range from −15 eV to 15 eV, the main contribution comes from the C-*p* and Si-*p* orbitals.

## 4. Conclusions

The structural, mechanical, anisotropic, and electronic properties of SiC_2_ and SiC_4_ have been investigated for the first time, utilizing first-principle calculations based on density functional theory. The elastic constants and phonon calculations reveal that SiC_2_ and SiC_4_ are mechanically and dynamically stable at ambient pressure. Moreover, by analyzing the *B*/*G* ratio, SiC_2_ and SiC_4_ are naturally brittle. The anisotropic calculations show that SiC_2_ and SiC_4_ are anisotropic materials and that SiC_4_ exhibits a greater anisotropy than SiC_2_. Finally, the band structure calculations predict that SiC_4_ is a quasi-direct band gap semiconductor, with a band gap of 2.28 eV, while SiC_2_ is an indirect band gap semiconductor, with a band gap of 0.91 eV.

## Figures and Tables

**Table 1:** The calculated lattice parameters and elastic moduli of SiC_2_, SiC_4_, and 3C-SiC. (Space group: SG).

Materials	SG	Methods	*a*	*b*	*C*	*β*	*B*	*G*	*E*	*v*
SiC_2_	*P*4_2_*nm*	PBE ^1^	4.197		7.107		203	162	384	0.18
		PBEsol ^1^	4.193		7.100		205	172	403	0.17
		CA-PZ ^1^	4.141		7.010		217	178	419	0.18
SiC_4_	*P*2_1_/*m*	PBE ^1^	6.755	2.763	4.379	75.78	285	258	595	0.15
		PBEsol ^1^	6.744	2.749	4.369	75.75	230	254	557	0.10
		CA-PZ ^1^	6.752	2.762	4.378	75.81	250	274	602	0.10
SiC	F-43m	PBE ^1^	4.348				217	187	436	0.17
		PBEsol ^1^	4.362				216	186	433	0.17
		CA-PZ ^1^	4.300				229	200	465	0.16
		PBE ^2^	4.380				235 ^5^			
		PBE ^3^	4.344				224 ^6^			
		Exp. ^4^	4.360				227 ^7^	192	448	0.17

^1^ This work, ^2^ Ref [10], ^3^ Ref [35], ^4^ Ref [36], ^5^ Ref [37], ^6^ Ref [38], ^7^ Ref [39].

**Table 2:** The calculated elastic constants of SiC_2_, SiC_4_, and 3C-SiC.

Materials	Methods	*C*_11_	*C*_22_	*C*_33_	*C*_44_	*C*_55_	*C*_66_	*C*_12_	*C*_13_	*C*_23_	*C*_15_	*C*_25_	*C*_35_	*C*_46_
SiC_2_	PBE ^1^	373		447	172		181	94	114					
	PBEsol ^1^	398		449	186		177	103	100					
	CA-PZ ^1^	409		483	191		191	101	115					
SiC_4_	PBE ^1^	606	650	648	316	280	196	58	188	87	−7	−9	−22	−19
	PBEsol ^1^	576	560	619	290	285	187	65	117	42	−16	3	−6	−11
	CA-PZ ^1^	609	612	677	313	305	203	59	121	54	−23	−1	−8	−15
SiC	PBE ^1^	385			243			132						
	PBEsol ^1^	381			244			133						
	CA-PZ ^1^	408			261			140						
	PBE ^2^	382			239			128						
	CA-PZ ^3^	390			253			134						
	Exp. ^4^	390			256			142						

^1^ This work, ^2^ Ref [10], ^3^ Ref [36], ^4^ Ref [40].
